# ZSM-5-SO_3_H: An Efficient Catalyst for Acylation of Sulfonamides Amines, Alcohols, and Phenols under Solvent-Free Conditions

**DOI:** 10.1155/2013/951749

**Published:** 2013-12-08

**Authors:** Ahmad Reza Massah, Roozbeh Javad Kalbasi, Mahdiehsadat Khalifesoltani, Fariba Moshtagh Kordesofla

**Affiliations:** ^1^Department of Chemistry, Islamic Azad University Shahreza Branch, Shahreza, Isfahan 86145-311, Iran; ^2^Razi Chemistry Research Center, Islamic Azad University Shahreza Branch, Shahreza, Isfahan 86145-311, Iran

## Abstract

Sulfonamides amines, alcohols, and phenols were efficiently acylated with carboxylic acid anhydrides and chlorides using ZSM-5-SO_3_H as catalyst under mild and solvent-free conditions. Also, direct esterification of alcohols with carboxylic acids occurred readily in the presence of this catalyst. Different types of amides and esters were obtained in moderate to high yields and purity after a simple workup. No chromatographic separation is needed for isolation of the acylated product. The catalyst was recovered and reused for up to four times without a noticeable decrease in catalytic activity.

## 1. Introduction

The acylation of alcohols, phenols, and amines is one of the most frequently used processes in organic chemistry. It provides an economical and efficient method for protecting hydroxyl groups during oxidation, peptide coupling, and glycosidation reactions [1]. Acylation is usually carried out by treatment of an alcohol or amine with carboxylic acid chlorides or anhydrides in the presence of an acid or a base catalyst in a suitable organic solvent. Basic catalysts such as 4-(dimethylamino) pyridine (DMAP) [2], tributylphosphines [3], 4-pyrrolidinopyridine [4], and acidic catalysts like Sc(OTf)_3_ [5], Gd(OTf)_3_ [6], lanthanide(III) tosylates [7], RuCl_3_ [8], Al(HSO_4_)_3_ [9]_,_ Bi(OTf)_3_ [10], and LiClO_4_ [11] catalyze acylation reactions with acid chloride or anhydride as the acylating agent under homogenous conditions. Use of homogenous catalysts poses serious problems, such as difficulty in the separation and recovery of the catalyst, disposal of the spent catalyst, and corrosion problems. Solid acid catalysts such as commercial zeolites [12] and montmorillonite K-10 or KSF clay [13] and ZnO [14] have been reported for the acylation of alcohols with acetic anhydride. Though acylation of alcohols can also be brought about by the action of Lewis acid reagents in conjunction with carboxylic acids, the Lewis acid is destroyed in the workup procedure resulting in substantial waste production [15]. Some heterogenized homogenous catalysts have also been reported for the acylation of alcohols and amines [16]. Most of the methods have some disadvantages, such as exothermic reaction, formation of by-products, complicated conditions, and excess acylating agents, and require longer reaction times, use of halogenated solvents, and expensive moisture-sensitive toxic reagents. Apart from these difficulties, some of the above methods do not satisfy the requirements of green synthesis due to the inability to recover and reuse the catalyst. Thus, due to high importance of the acylation reactions, development of easily separable and reusable solid catalyst having high activity for the acylation reaction is of great practical importance.

The use of reusable heterogeneous solid acid catalysts received much attention because of their special advantages such as stability (toward air and moisture), lack of corrosion, ease of handling, recovery, low waste generation, and environmental friendliness, such as zeolites, clays and heteropolyacids [17]. Among zeolites, ZSM-5 is an aluminosilicate zeolite and is composed of several pentasil units linked together by oxygen bridges to form pentasil chain. ZSM-5 has high silicon to aluminum ratio; whenever an Al^3+^ cation replaces a Si^4+^ cation, an addition of positive charge is required to keep the material charge neutral with proton as the cation and the material becomes very acidic. The very regular 3D structure and the acidity of ZSM-5 can be utilized for acid-catalyzed reactions [18].

**Scheme 1:**
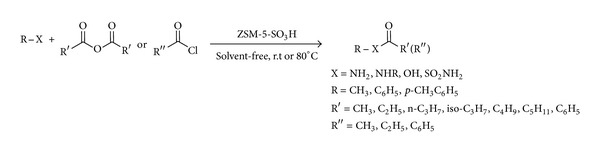


**Scheme 2:**
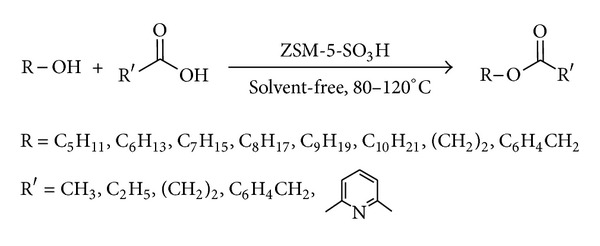


Recently, as a part of our ongoing research project to develop newer environmentally benign synthetic methodologies using solid acid catalyst [19–22], ZSM-5-SO_3_H was synthesized for the first time in our group and was used in the acylation of aldehydes [23] and Mannich reaction [24]. Based on our previous works on solvent-free reactions particularly in acylation reactions [25–28], herein we wish to report our results on the acylation of alcohols, phenols, aliphatic and aromatic amines, and sulfonamides with some carboxylic acid anhydrides and chlorides (Scheme 1) and also acylation of alcohols with carboxylic acids (Scheme 2) under solvent-free conditions.

## 2. Experimental

All chemicals were purchased from Merck and Fluka chemical companies. Infrared spectra were recorded on a Perkin-Elmer V IR spectrophotometer. ^1^HNMR and ^13^CNMR spectra were recorded on a Bruker (400 MHz) spectrometer in CDCl_3_. All reactions were conducted open to the atmosphere, and the yields refer to isolated products. The products were characterized by comparison of their spectral and physical data with those of authentic samples. ZSM-5 and ZSM-5-SO_3_H were synthesized following the procedure previously reported [19].

### 2.1. General Procedure for Acylation of Amines

Amine, (2 mmol) and ZSM-5-SO_3_H (0.01 g) were ground altogether into fine powder, and carboxylic acid anhydride (2 mmol benzoic anhydride, 4 mmol acetic anhydride, or 2.2 mmol of other anhydride) or carboxylic acid chloride (2 mmol) was added under vigorous stirring at low temperature in an ice bath and then was stirred at room temperature. The progress of the reaction was monitored by TLC. Upon completion of the reaction, CH_2_Cl_2_ (20 mL) was added and the catalyst was filtered and washed with additional solvent (10 mL). The filtrate was washed with NaHCO_3_ (5%, 10 mL) water (10 mL) and dried over anhydrous Na_2_SO_4_, and the solvent was evaporated to yield the product. The products were obtained in high purity (>95%).

### 2.2. General Procedure for Acylation of Sulfonamides

Sulfonamide (1 mmol) and ZSM-5-SO_3_H (0.01 g) were ground altogether into fine powder, and carboxylic acid anhydride (1.5 mmol) was added under vigorous stirring at room temperature. The progress of the reaction was monitored by TLC. Upon completion of the reaction, the products were obtained as described in previous procedure in high yield and purity.

### 2.3. General Procedure for Acylation of Alcohols and Phenols

Alcohol (1 mmol) and ZSM-5-SO_3_H (0.01–0.05 g) were ground altogether into fine powder, and carboxylic acid anhydride (1 mmol benzoic anhydride, 2 mmol acetic anhydride, or 1.2 mmol of other anhydride) or carboxylic acid chloride (1.2 mmol) was added under vigorous stirring at room temperature (for acetic anhydride), 80°C (for benzoic anhydride and benzoyl chloride), or 50°C (for other anhydrides). The progress of the reaction was monitored by TLC. Upon completion of the reaction, the products were obtained as described in previous procedure in high yield and purity.

### 2.4. General Procedure for Acylation of Alcohols with Carboxylic Acids

Alcohol (1 mmol) and ZSM-5-SO_3_H (0.025–0.1 g) were ground altogether into fine powder, and carboxylic acid or dicarboxylic acid (1.2–2.4 mmol) was added under vigorous stirring at 80–120°C. The progress of the reaction was monitored by TLC. Upon completion of the reaction, CH_2_Cl_2_ (30 mL) was added and the catalyst was filtered and washed with additional solvent (10 mL). The filtrate was washed with NaHCO_3_ (5%, 30 mL) water (20 mL) dried over anhydrous Na_2_SO_4_ and the solvent was evaporated to yield the product. The products were obtained in high purity (>95%).

#### 2.4.1. Spectral Data of Some Isolated Products

*N*-(2-Methylphenyl) benzamide (Table 2, entry 5): m.p = 265°C, FT-IR (KBr, cm^−1^): 3242, 3055, 1649, 1604, 1525, 1488, 1307, 748, 712; ^1^H NMR (400 MHz, CDCl_3_), *δ* (ppm): 2.34 (s, 3H), 7.15 (t, 1H, *J* = 7.2 Hz), 7.25 (dd, 2H, *J*_1_ = 7.6, *J*_2_ = 7.6 Hz), 7.50 (dd, 2H, *J*_1_ = 7.6, *J*_2_ = 7.6 Hz), 7.58 (t, 1H, *J* = 7.2 Hz), 7.94 −7.86 (m, 4H); ^13^C NMR (100 MHz, CDCl_3_), *δ* (ppm): 17.9, 123.3, 125.4, 126.9, 127.1, 128.8, 129.4, 130.6, 131.9, 135.0, 135.8, 165.7.

*N*-(2,4-Dimethylphenyl) benzamide (Table 2, entry 6): m.p = 192°C, FT-IR (KBr, cm^−1^): 3266, 3023, 1648, 1602, 1517, 1308, 1279, 817, 709; ^1^H NMR (400 MHz, CDCl_3_), *δ* (ppm): 2.32 (s, 3H), 2.35 (s, 3H), 7.06–7.11 (m, 2H), 7.52–7.59 (m, 3H), 7.68 (br, 1H), 7.77 (d, 1H, *J* = 7.6 Hz), 7.91 (d, 2H, *J* = 7.6 Hz); ^13^C NMR (100 MHz, CDCl_3_), *δ* (ppm): 17.8, 20.9, 123.6, 127.1, 127.4, 128.8, 129.8, 131.3, 131.7, 133.1, 135.1, 135.2, 165.8.

*N*-(3-Chlorophenyl) benzamide (Table 2, entry 8): m.p = 120°C, FT-IR (KBr, cm^−1^): 3296, 3072, 1651, 1591, 1522, 1419, 1294, 1250, 777, 700; ^1^H NMR (400 MHz, CDCl_3_), *δ* (ppm): 7.12–7.26 (m, 2H), 7.43–7.53 (m, 4H), 7.79–7.84 (m, 3H), 8.31 (s, 1H); ^13^C NMR (100 MHz, CDCl_3_), *δ* (ppm): 118.5, 120.6, 124.6, 127.1, 128.8, 130.0, 132.1, 134.5, 134.6, 139.1, 166.2.

*N*-(3-Nitrophenyl) benzamide (Table 2, entry 9): m.p = 157°C, FT-IR (KBr, cm^−1^): 3361, 3065, 1662, 1593, 1529, 1417, 1351, 1299, 799, 706; ^1^H NMR (400 MHz, CDCl_3_), *δ* (ppm): 7.63–7.51 (m, 4H), 7.92 (d, 2H, *J* = 7.2 Hz), 8.03 (d, 1H, *J* = 8.0 Hz), 8.13 (d, 2H, *J* = 8.8 Hz), 8.53 (s, 1H); ^13^C NMR (100 MHz, CDCl_3_), *δ* (ppm): 115.0, 119.1, 125.9, 127.1, 129.0, 130.0, 132.5, 134.0, 139.1, 148.7, 166.0.

*N*-Hexyl benzamide (Table 2, entry 11): m.p = 40°C, FT-IR (KBr, cm^−1^): 3342, 3080, 2924, 2856, 1631, 1576, 1528, 1483, 1310, 1273, 717, 694; ^1^H NMR (400 MHz, CDCl_3_), *δ* (ppm): 0.88 (t, 3H, *J* = 6.8 Hz), 1.40–1.24 (m, 6H), 1.59 (quint, 2H, *J* = 7.2 Hz), 3.40 (q, 2H, *J* = 6.8 Hz), 6.76 (br, 1H), 7.38–7.46 (m, 3H), 7.79 (d, 2H, *J* = 7.2 Hz); ^13^C NMR (400 MHz, CDCl_3_), *δ* (ppm): 14.0, 22.6, 26.7, 29.6, 31.5, 40.2, 127.0, 128.4, 131.2, 134.9, 167.7.

*N*, *N*-(Diisopropyl) benzamide (Table 2, entry 12): FT-IR (KBr, cm^−1^): 3076, 2969, 1627, 1444, 1373, 1340, 1211, 1158, 779, 705; ^1^H NMR (400 MHz, CDCl_3_), *δ* (ppm): 1.20 (br, 6H), 1.40 (br, 6H), 3.68 (br, 1H), 3.87 (br, 1H), 7.34−7.29 (m, 2H), 7.41–7.34 (m, 3H).

*N*-(4-Methylphenyl) acetamide (Table 2, entry 16): m.p = 154°C, FT-IR (KBr, cm^−1^): 3293, 3065, 1663, 1605, 1551, 1508, 1448, 1401, 1365, 1320, 1263, 821, 753, 508; ^1^H NMR (400 MHz, CDCl_3_), *δ* (ppm): 2.16 (s, 3H), 2.33 (s, 3H), 7.12 (d, 2H, *J* = 8.4 Hz), 7.40 (d, 2H, *J* = 8.4 Hz), 7.80 (br, 1H); ^13^C NMR (100 MHz, CDCl_3_), *δ* (ppm): 20.9, 24.4, 120.2, 129.4, 133.9, 135.5, 168.7.

*N*, *N*-(Dibenzyl) acetamide (Table 2, entry 18): FT-IR (KBr, cm^−1^): 3061, 2926, 1648, 1421, 1239, 734, 699; ^1^H NMR (400 MHz, CDCl_3_), *δ* (ppm): 2.25 (s, 3H), 4.47 (s, 2H), 4.63 (s, 2H), 7.20 (d, 2H, *J* = 7.2 Hz), 7.26 (d, 2H, *J* = 6.8 Hz), 7.29–7.37 (m, 4H), 7.41 (t, 2H, *J* = 7.2 Hz); ^13^C NMR (100 MHz, CDCl_3_), *δ* (ppm): 21.7, 48.0, 50.8, 126.5, 127.5, 127.7, 128.3, 128.6, 129.0, 136.5, 137.4, 171.1.

*N*-(4-Methylphenylsulfonyl) acetamide (Table 2, entry 29): FT-IR (KBr, cm^−1^): 3293, 1720, 1441, 1331, 1213, 1000, 866, 667, 537; ^1^H NMR (400 MHz, CDCl_3_), *δ* (ppm): 1.90 (s, 3H), 2.37 (s, 3H), 7.40 (d, 2H, *J* = 7.9 Hz), 7.79 (d, 2H, *J* = 8.1 Hz), 12.01 (s, 1H); ^13^C NMR (100 MHz, CDCl_3_), *δ* (ppm): 21.5, 32.6, 128.0, 130.0, 136.9, 144.6, 169.1.

*N*-(4-Methylphenylsulfonyl) pentanamide (Table 2, entry 30): FT-IR (KBr, cm^−1^): 3251, 2953, 2871, 1711, 1446, 1342, 1167, 1082, 855, 748, 663, 551; ^1^H NMR (400 MHz, CDCl_3_), *δ* (ppm): 0.77 (t, 3H, *J* = 7.2 Hz), 1.15 (sext, 2H, *J* = 7.4 Hz), 1.37 (quint, 2H, *J* = 7.1 Hz), 2.18 (t, 2H, *J* = 7.2 Hz), 2.37 (s, 3H), 3.39 (d, 2H, *J* = 8.1 Hz), 7.79 (d, 2H, *J* = 8.2 Hz), 11.96 (s, 1H); ^13^C NMR (100 MHz, CDCl_3_), *δ* (ppm): 13.9, 21.5, 21.8, 26.5, 35.5, 127.9, 129.9, 137.1, 145.0, 171.9.

n-Butyl benzoate (Table 3, entry 1): FT-IR (KBr, cm^−1^): 3066, 2960, 2873, 1720, 1602, 1453, 1275, 1110, 711; ^1^H NMR (400 MHz, CDCl_3_), *δ* (ppm): 1.01 (t, 3H, *J* = 7.2 Hz), 1.51 (sext, 2H, *J* = 7.6 Hz), 1.78 (quint, 2H, *J* = 7.2 Hz), 4.36 (t, 2H, *J* = 6.8 Hz), 7.46–7.57 (m, 3H), 8.08 (d, 2H, *J* = 7.2 Hz); ^13^C NMR (100 MHz, CDCl_3_), *δ* (ppm): 13.8, 19.3, 30.8, 64.8, 128.3, 129.6, 130.6, 132.8, 166.7.

n-Pentyl benzoate (Table 3, entry 2): FT-IR (KBr, cm^−1^): 3067, 2958, 2862, 1720, 1602, 1453, 1274, 1110, 711; ^1^H NMR (400 MHz, CDCl_3_), *δ* (ppm): 0.93 (t, 3H, *J* = 7.2 Hz), 1.49–1.32 (m, 4H), 1.76 (quint, 2H, *J* = 6.8 Hz), 4.31 (t, 2H, *J* = 6.8 Hz), 7.41-7.51 (m, 3H), 8.06 (d, 2H, *J* = 7.6 Hz); ^13^C NMR (100 MHz, CDCl_3_), *δ* (ppm): 14.0, 22.4, 28.2, 28.4, 53.5, 65.1, 128.3, 129.5, 130.5, 132.8, 166.7.

n-Hexyl benzoate (Table 3, entry 3): FT-IR (KBr, cm^−1^): 3066, 2956, 2931, 2860, 1721, 1602, 1453, 1274, 1111, 711; ^1^H NMR (400 MHz, CDCl_3_), *δ* (ppm): 0.94 (t, 3H, *J* = 7.2 Hz), 1.51–1.30 (m, 6H), 1.80 (quint, 2H, *J* = 6.8 Hz), 4.35 (t, 2H, *J* = 6.8 Hz), 7.46–7.58 (m, 3H), 8.08 (d, 2H, *J* = 7.2 Hz); ^13^C NMR (100 MHz, CDCl_3_), 166.7, 132.8, 130.6, 129.5, 128.3, 65.1, 31.5, 28.7, 25.7, 22.6, 14.0

n-Decyl benzoate (Table 3, entry 5): FT-IR (KBr, cm^−1^): 3068, 2926, 2856, 1721, 1602, 1457, 1274, 1111, 711; ^1^H NMR (400 MHz, CDCl_3_), *δ* (ppm): 0.91 (t, 3H, *J* = 7.2 Hz), 1.52–1.25 (m, 14H), 1.80 (quint, 2H, *J* = 7.2 Hz), 4.35 (t, 2H, *J* = 6.8 Hz), 7.47–7.58 (m, 3H), 8.07 (d, 2H, *J* = 7.2 Hz); ^13^C NMR (100 MHz, CDCl_3_), *δ* (ppm): 14.1, 22.7, 26.1, 28.8, 29.3, 29.4, 29.6, 31.9, 65.1, 128.3, 129.5, 130.6, 132.8, 166.6.

n-Hexyl acetate (Table 3, entry 7): FT-IR (KBr, cm^−1^): 2957, 2933, 1742, 1462, 1367, 1240, 1039; ^1^H NMR (400 MHz, CDCl_3_), *δ* (ppm): 0.84 (t, 3H, *J* = 6.8 Hz), 1.35–1.20 (m, 6H), 1.56 (quint, 2H, *J* = 6.8 Hz), 1.99 (s, 3H), 4.01 (t, 2H, *J* = 6.8 Hz); ^13^C NMR (100 MHz, CDCl_3_), *δ* (ppm): 13.9, 20.9, 22.5, 25.6, 28.6, 31.4, 64.6, 171.2.

n-Octyl acetate (Table 3, entry 8): FT-IR (KBr, cm^−1^): 2929, 2859, 1743, 1463, 1367, 1239, 1040; ^1^H NMR (400 MHz, CDCl_3_), *δ* (ppm): 0.86 (t, 3H, *J* = 6.8 Hz), 1.38–1.21 (m, 10H), 1.60 (m, 2H), 2.03 (s, 3H), 4.04 (t, 2H, *J* = 6.8 Hz); ^13^C NMR (400 MHz, CDCl_3_), *δ* (ppm): 14.0, 20.9, 22.6, 25.9, 28.6, 29.1, 29.2, 31.8, 64.6, 171.1.

n-Decyl acetate (Table 3, entry 9): FT-IR (KBr, cm^−1^): 2927, 2858, 1743, 1464, 1366, 1239, 1041; ^1^H NMR (400 MHz, CDCl_3_), *δ* (ppm): 0.87 (t, 3H, *J* = 6.8 Hz), 1.39–1.21 (m, 14H), 1.60 (quint, 2H, *J* = 6.8 Hz), 2.03 (s, 3H), 4.04 (t, 2H, *J* = 6.8 Hz); ^13^C NMR (100 MHz, CDCl_3_), *δ* (ppm): 14.0, 20.9, 22.6, 25.9, 28.6, 29.2, 29.3, 29.5, 31.9, 64.6, 171.1.

Benzyl acetate (Table 3, entry 12): FT-IR (KBr, cm^−1^): 3034, 2954, 1742, 1455, 1380, 1230, 1027, 747, 698; ^1^H NMR (400 MHz, CDCl_3_), *δ* (ppm): 2.14 (s, 3H), 5.16 (s, 2H), 7.43–7.35 (m, 5H); ^13^C NMR (100 MHz, CDCl_3_), *δ* (ppm): 21.0, 66.3, 76.9, 77.2, 77.5, 128.3, 128.3, 128.6, 136.0, 170.9.

n-Octyl-4-methylbenzoate (Table 3, entry 23): FT-IR (KBr, cm^−1^): 1719, 1612, 1464, 1274, 1107, 752; ^1^H NMR (400 MHz, CDCl_3_), *δ* (ppm): 0.92 (t, 3H, *J* = 6.8 Hz), 1.27–1.42 (m, 10H), 1.79 (quint, 2H, *J* = 6.8 Hz), 2.44 (s, 3H), 4.33 (t, 2H, *J* = 6.8 Hz), 7.26 (d, 1H, *J* = 8.0 Hz), 7.96 (d, 1H, *J* = 8.0 Hz); ^13^C NMR (100 MHz, CDCl_3_), *δ* (ppm): 14.1, 21.6, 22.7, 26.1, 28.8, 29.2, 29.3, 31.8, 64.9, 76.7, 77.0, 77.4, 127.9, 129.0, 129.6, 143.4, 166.8.

n-Hexyl-4-methylbenzoate (Table 3, entry 24): FT-IR (KBr, cm^−1^): 1719.3, 1612.6, 1274.2, 1106.9, 753.9; ^1^H NMR (400 MHz, CDCl_3_), *δ* (ppm): 0.93 (t, 3H, *J* = 7.2 Hz), 1.33–1.42 (m, 6H), 1.79 (quint, 2H, *J* = 6.8 Hz), 2.44 (s, 3H), 4.33 (t, 2H, *J* = 6.4 Hz), 7.26 (d, 2H, *J* = 8.0 Hz), 7.96 (d, 2H, *J* = 8.0 Hz); ^13^C NMR (100 MHz, CDCl_3_), *δ* (ppm): 14.0, 21.7, 22.6, 25.7, 28.7, 31.5, 65.0, 77.0, 77.2, 127.8, 129.04, 129.6, 143.4, 166.8.

Decyl propanoate (Table 3, entry 29): IR (KBr, cm^−1^): 1740, 1464, 1186, 1083, 749; ^1^H NMR (400 MHz, CDCl_3_): *δ* (ppm): 0.89 (t, 3H, *J* = 6.4 Hz), 1.15 (t, 3H, *J* = 7.6 Hz), 1.22–1.38 (m, 14H), 1.58–1.67 (m, 2H), 2.33 (q, 2H, *J* = 7.6 Hz), 4.07 (t, 2H, *J* = 6.8 Hz); ^13^C NMR (100 MHz, CDCl_3_): *δ* (ppm): 9.2, 14.1, 22.7, 25.9, 27.6, 28.7, 29.3, 29.3, 29.5, 31.9, 64.5, 174.6.

Octyl propanoate (Table 3, entry 30): IR (KBr, cm^−1^): 1740, 1464, 1186, 1083, 723; ^1^H NMR (400 MHz, CDCl_3_): *δ* (ppm): 0.86 (t, 3H, *J* = 6.4 Hz), 1.12 (t, 3H, *J* = 7.6 Hz), 1.22–1.38 (m, 10H), 1.59 (quint, 2H, *J* = 7.2 Hz), 2.30 (q, 2H, *J* = 7.6 Hz), 4.04 (t, 2H, *J* = 6.8 Hz); ^13^C NMR (100 MHz, CDCl_3_): *δ* (ppm): 9.1, 14.0, 22.6, 25.9, 27.6, 28.6, 29.2, 29.2, 31.8, 64.4, 174.5.

Ethane-1,2-diylbis(2-phenylacetate) (Table 3, entry 31): IR (KBr, cm^−1^): 1738, 1454, 1245, 1139, 726; ^1^H NMR (400 MHz, CDCl_3_): *δ* (ppm): 3.64 (s, 4H), 4.31–4.36 (m, 4H), 7.28–7.40 (m, 10H), ^13^C NMR (100 MHz, CDCl_3_): *δ* (ppm): 41.1, 62.4, 127.2, 128.6, 129.3, 133.8, 171.3.

n-Heptyl phenylacetate (Table 3, entry 32): IR (KBr, cm^−1^): 1737, 1456, 1251, 1155, 721; ^1^H NMR (400 MHz, CDCl_3_): *δ* (ppm): 0.95 (t, 3H, *J* = 6.4 Hz), 1.26–1.41 (m, 8H), 1.61–1.72 (m, 2H), 3.67 (s, 2H), 4.14 (t, 2H, *J* = 6.4 Hz), 7.40–7.28 (m, 5H); ^13^C NMR (100 MHz, CDCl_3_): *δ* (ppm): 14.1, 22.6, 25.8, 28.6, 28.9, 31.8, 41.5, 65.0, 76.8, 77.2, 77.5, 127.0, 128.6, 129.3, 134.3, 171.7.

n-Octyl phenylacetate (Table 3, entry 33): IR (KBr, cm^−1^): 1737, 1456, 1252, 1154, 721.2; ^1^H NMR (400 MHz, CDCl_3_): *δ* (ppm): 0.95 (t, 3H, *J* = 6.8 Hz), 1.24–1.41 (m, 10H), 1.62–1.71(m, 2H), 3.67 (s, 2H), 4.14 (t, 2H, *J* = 6.4 Hz), 7.40–7.27 (m, 5H); ^13^C NMR (100 MHz, CDCl_3_): *δ* (ppm): 14.1, 22.7, 25.9, 28.6, 29.2, 31.8, 41.5, 65.0, 76.8, 77.1, 77.4, 127.0, 128.6, 129.3, 134.3.

n-Decyl phenylacetate (Table 3, entry 34): IR (KBr, cm^−1^): 1738, 1456, 1251, 1155, 721; ^1^H NMR (400 MHz, CDCl_3_): *δ* (ppm): 0.93 (t, 3H, *J* = 6.4 Hz), 1.27–1.38 (m, 14H), 1.61–1.68 (m, 2H), 3.66 (s, 2H), 4.12 (t, 2H, *J* = 6.8 Hz), 7.39–7.27 (m, 5H); ^13^C NMR (100 MHz, CDCl_3_): *δ* (ppm): 14.2, 22.7, 25.9, 28.6, 29.2, 29.3, 29.5, 31.9, 41.5, 65.1, 76.8, 77.1, 77.4, 127.0, 128.6, 129.3, 134.3.

Dipentylpyridine-2,6-dicarboxylate (Table 3, entry 35): IR (KBr, cm^−1^): 1741, 1465, 1244.0, 1149, 759; ^1^H NMR (400 MHz, CDCl_3_): *δ* (ppm): 0.94 (t, 6H, *J* = 8.0 Hz), 1.36–1.50 (m, 8H), 1.85 (quint, 4H, *J* = 6.8 Hz), 4.43 (t, 4H, *J* = 6.8 Hz), 8.02 (t, 1H, *J* = 7.6 Hz), 8.28 (d, 2H, *J* = 8.0 Hz); ^13^C NMR (100 MHz, CDCl_3_): *δ* (ppm): 14.0, 22.4, 28.1, 28.3, 66.4, 76.7, 77.1, 77.3, 77.4, 127.7, 138.1, 148.7 164.7.

Dihexylpyridine-2,6-dicarboxylate (Table 3, entry 36): IR (KBr, cm^−1^): 1722, 1241, 1144, 753; ^1^H NMR (400 MHz, CDCl_3_): *δ* (ppm): 0.82–0.93 (m, 6H) 1.81 (quint, 4H, *J* = 7.2 Hz), 1.22–1.38 (m, 8H), 1.38-1.48 (m, 4H), 4.39 (t, 4H, *J* = 7.2 Hz), 7.99 (t, 1H, *J* = 8.0 Hz), 8.24 (d, 2H, *J* = 7.6 Hz).

Diheptyl glutarate (Table 3, entry 37): IR (KBr, cm^−1^): 1738, 1465, 1148, 1065, 725; ^1^H NMR (400 MHz, CDCl_3_): *δ* (ppm) 0.88 (t, 6H, *J* = 6.8 Hz), 1.22–1.38 (m, 16H), 1.60 (quint, 4H, *J* = 6.8 Hz), 1.95 (quint, 4H, *J* = 7.2 Hz), 2.37 (t, 4H, *J* = 7.2 Hz), 4.06 (t, 4H, *J* = 6.8 Hz); ^13^C NMR (100 MHz, CDCl_3_): *δ* (ppm) 14.0, 20.2, 22.6, 25.9, 28.6, 28.9, 31.7, 33.4, 64.6, 173.1.

Dioctyl glutarate (Table 3, entry 38): IR (KBr, cm^−1^): 1737, 1465, 1064, 722; ^1^H NMR (400 MHz, CDCl_3_): *δ* (ppm): 0.90 (t, 6H, *J* = 6.4 Hz), 1.24–1.40 (m, 20H), 1.59–1.68 (m, 4H), 1.92–2.02 (m, 4H), 2.36–2.48 (m, 4H), 4.08 (t, 4H, *J* = 5.6 Hz); ^13^C NMR (100 MHz, CDCl_3_): *δ* (ppm): 14.1, 19.9, 20.2, 22.6, 25.9, 28.6, 29.2, 29.2, 31.8, 33.0, 33.2, 33.4, 64.7, 64.7, 173.0, 173.1.

## 3. Results and Discussion

As a starting point for this work, the reaction was optimized in order to find the best conditions especially in agreement with green chemistry (Table 1). To find the optimum conditions for acylation of amines and sulfonamides with carboxylic acid anhydrides and chlorides, several sets of reaction conditions were examined (Table 1, entries 1–5). Thus, under the best conditions, amines (2 mmol) were acylated at room temperature almost quantitatively with carboxylic acid anhydrides or chlorides (2–4 mmol) in the presence of 0.005–0.01 g of ZSM-5-SO_3_H without use of any solvents (Table 1, entries 1–4). Acylation of sulfonamides (1 mmol) occurred only with aliphatic anhydrides (1.5 mmol) in the presence of 0.01 g of catalyst at room temperature under solvent-free conditions (Table 1, entry 5).

Then, the reaction was explored with a variety of aromatic and aliphatic amines to evaluate the scope and limitations of this method (Table 2). The results showed that the different aromatic amines containing various electron-donating and -withdrawing groups as well as aliphatic amines reacted with carboxylic acid chlorides or anhydrides within 5–30 minutes to produce the corresponding amides in 70%–95% yield. Benzoic anhydride and benzoyl chloride was used as aromatic acylating agents. Also, acylation of amines were examined using different aliphatic acylating agents including acetic, pentanoic, and isobutanoic anhydrides. The excellent activity of ZSM-5-SO_3_H was demonstrated by the good to high yields obtained for anilines having electron-withdrawing groups such as Cl and NO_2_ (Table 2, entries 7–9). However, the best yields were obtained with substrates bearing electron-donating groups, such as methoxy especially in the paraposition (Table 2, entries 2 and 14). Also very good results were obtained when secondary amines were acylated in the presence of ZSM-5-SO_3_H, and the corresponding amides were obtained in 78%–90% yields in 10–30 minutes at room temperature under solvent-free conditions (Table 2, entries 12 and 18). Finally, this method was used for the acylation of 4-methylbenzenesulfonamide as a weak nucleophile with acetic and pentanoic anhydrides. Interestingly, the corresponding *N*-acyl sulfonamides were obtained in 80% and 92% yields after 5 and 15 minutes in high purity (Table 2, entries 29 and 30).

To demonstrate the versatility of this protocol, the acylation of alcohols and phenols was investigated using carboxylic acids, carboxylic acid anhydrides and chlorides, as acylating agents. At first, several sets of reaction conditions were examined to find the best conditions for acylation of alcohols. In the optimum conditions, alcohols and phenols (1 mmol) were acylated at room temperature with acetic anhydrides or chlorides (2 mmol) in the presence of 0.01 g of ZSM-5-SO_3_H under the solvent-free conditions (Table 1, entry 6). Acylation with benzoic anhydride was done at 80°C using 0.05 g of catalyst (Table 1, entry 7). When carboxylic acids were used as acylating agents, the reactions were done at 80–120°C (Table 1, entries 8–10).

Using a simple experimental procedure, the ZSM-5-SO_3_H catalyzed acylation of benzylic, primary and secondary alcohols proceeded efficiently using carboxylic acid anhydrides and chlorides (Table 3, entries 1–15). Different carboxylic acid anhydrides, and chlorides such as benzoic anhydride, acetic anhydride, pentanoic anhydride and acetyl chloride were used as acylating agent, and the corresponding esters were obtained in high isolated yields and purity after short reaction times under the solvent-free conditions. Primary alcohols were acylated faster than secondary ones (Table 3, entries 4), and sterically hindered alcohols, such as tert-butyl alcohol, remained unchanged. It was interesting to note that phenols were also satisfactorily acylated generating the corresponding esters in 75%–95% yields (Table 3, entries 13–15).

In order to generalize the catalytic efficiency of ZSM-5-SO_3_H for direct acylating with carboxylic acids, the acylation of alcohols was tried with different carboxylic acid (Table 3, entries 16–38) while most literature methods for direct acylation of alcohols employ only acetic acid [29–31]. Acylation with acetic, propanoic, phenyl acetic, benzoic acid, and 4-methylbenzoic acid resulted in good yield of the esters under the solvent-free conditions. Also pleasing results were obtained when acylation of alcohols was done with dicarboxylic acids such as glutaric acid and pyridine-1,6-dicarboxylic acid, and the corresponding diester was obtained in high purity in absence of solvent (Table 3, entries 35–38). It is noteworthy that, in contrast to most reported methods that need excess of acetic acid, in this method acylation of alcohols, was carried out using only 1.2–2.0 eq. of carboxylic acid. Also, acylation of ethylene glycol as a diol, was carried out with phenylacetic acid under solvent-free conditions, and the corresponding ester was obtained in 84% after 5 minutes. It should be mentioned that several efforts to use this method for direct acylation of amines for the synthesis of amides were not successful.

**Scheme 3:**
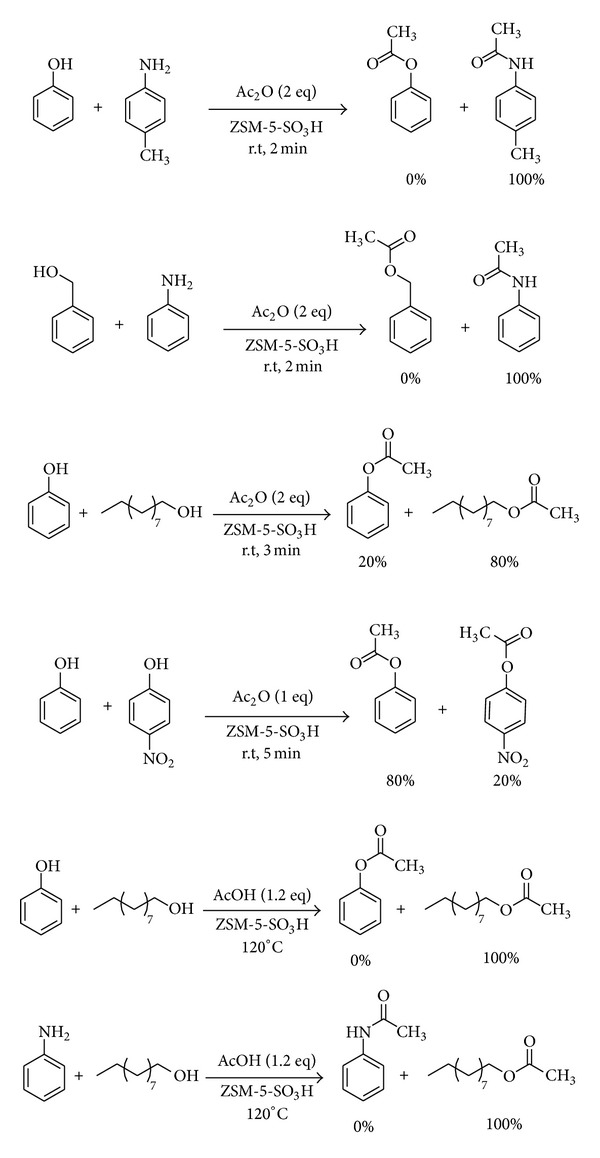


The importance of selectivity in organic chemistry encouraged us to consider the selectivity of acylation of different alcohols and amines. Several reactions were carried out using carboxylic acid anhydrides and chlorides as acylating agents, and, surprisingly, the excellent selectivity was found. For example, aromatic amines such as 4-methyl aniline can be converted into the amide in the presence of phenol. On the other hand, aromatic amines were acylated selectively in the presence of aliphatic alcohol. Furthermore, the reactions of alcohols with acylating agent were so fast, in comparison to those of the phenol, that the selective acylation of aliphatic alcohols in the presence of phenols appeared to be a distinct possibility. The result showed that selective acylation of phenols with or without electron-donating group in the presence of phenols with electron-withdrawing group is possible with this method. As well as carboxylic acid anhydrides and chlorides, the excellent selectivity was observed when carboxylic acids were used as acylating agent. Acylation of alcohols was carried out selectively in the presence of phenols. Also acylation of alcohols was done in the presence of amines selectively, while as mentioned above this selectivity is reversed when carboxylic acid anhydrides were used as acylating agent (Scheme 3).

To show another advantage of the present acylation method and the importance of scale-up ability for laboratory and industrial purposes, a few amines and phenols were acylated in large scale successfully without considerable limitation. For example, acylation of 4-methyl aniline (Table 2, entry 4), 1-decanol with benzoic anhydride (Table 3, entry 5), and 1-heptanol with benzoic acid (Table 3, entry 18) was carried out in 20 mmol scales as well as the 1 mmol ones. The only difference is the reaction time. Reaction in large scale needs more time, that is, because of the difficulty in stirring the reaction mixture under solvent-free conditions.

Finally, we were interested to study the reusability of the catalysts due to economical and environmental aspects. For this purpose, the reaction of 1-heptanol with benzoic anhydride and benzoic acid was chosen as model reactions. At the end of each run, the catalyst was recovered from the reaction mixture by addition of dichloromethane, simple filtration, and drying at 100°C and then reused. The recycled ZSM-5-SO_3_H was used for further runs, and its activity did not show any significant decrease even after four runs.

## 4. Conclusion

In conclusion, we have described a highly efficient and chemoselective synthetic route for the acylation of sulfonamides amines, alcohols, and phenols with carboxylic acids, carboxylic acid chlorides, and anhydrides in the presence of ZSM-5-SO_3_H under solvent-free conditions. This method has advantages in terms of low cost and nontoxic nature of the catalyst, high yield and purity of the products, short reaction times, operational simplicity, and easy workup. In addition, recyclability of this protocol is attractive and useful. Direct acylation of alcohols with different carboxylic acids is another advantage of this protocol.

## Figures and Tables

**Table 1:** The best conditions for ZSM-5-SO_3_H catalyzed acylation of alcohols and amines.

Entry	Amine or alcohol (mmol)	Acylating agent (mmol)	ZSM-5-SO_3_H (g)	Temperature (°C)
1	Amine (2)	Benzoic anhydride (2)	0.01	r.t
2	Amine (2)	Acetic anhydride (4)	0.01	r.t
3	Amine (2)	Aliphatic anhydride (2.2)	0.01	r.t
4	Amine (2)	Benzoyl chloride (2)	0.005	r.t
5	Sulfonamide (1)	Aliphatic anhydride (1.5)	0.01	r.t
6	Alcohol (1)^a^	Acetic anhydride or chloride (2)	0.01	r.t
7	Alcohol (1)^a^	Benzoic anhydride (1)	0.05	80
8	Alcohol (1)	Carboxylic acid^b^ (1.2)	0.025	100
9	Alcohol (2)	Dicarboxylic acid (4.5)	0.05	100
10	Diol (1)	Carboxylic acid (5)	0.1	120

^a^Phenol was acylated the same as alcohols.

^b^Alcohols were acylated with acetic acid at 80°C in the presence of 0.05 g of catalyst.

**Table 2:** ZSM-5-SO_3_H catalyzed acylation of amines under solvent-free conditions.

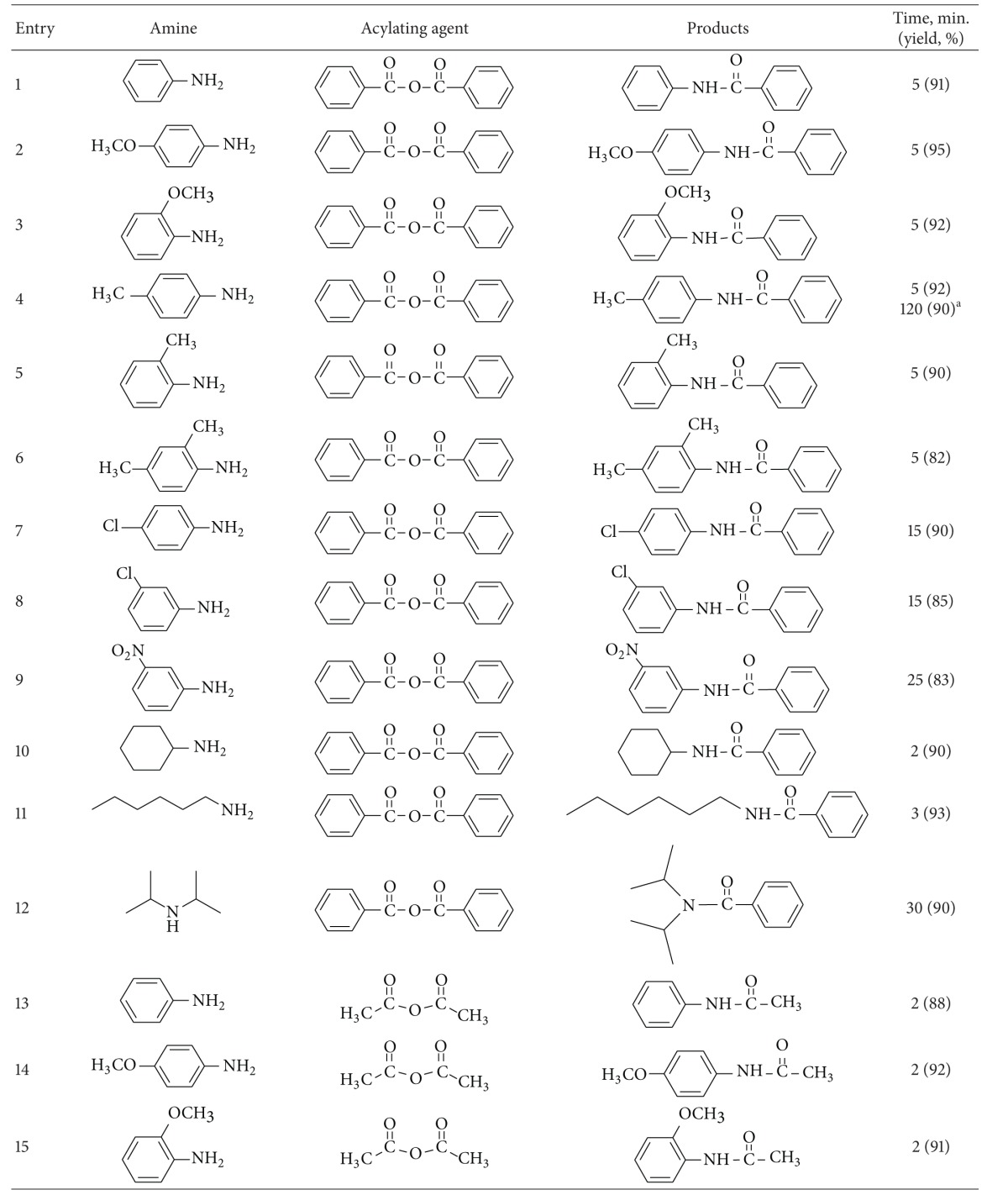 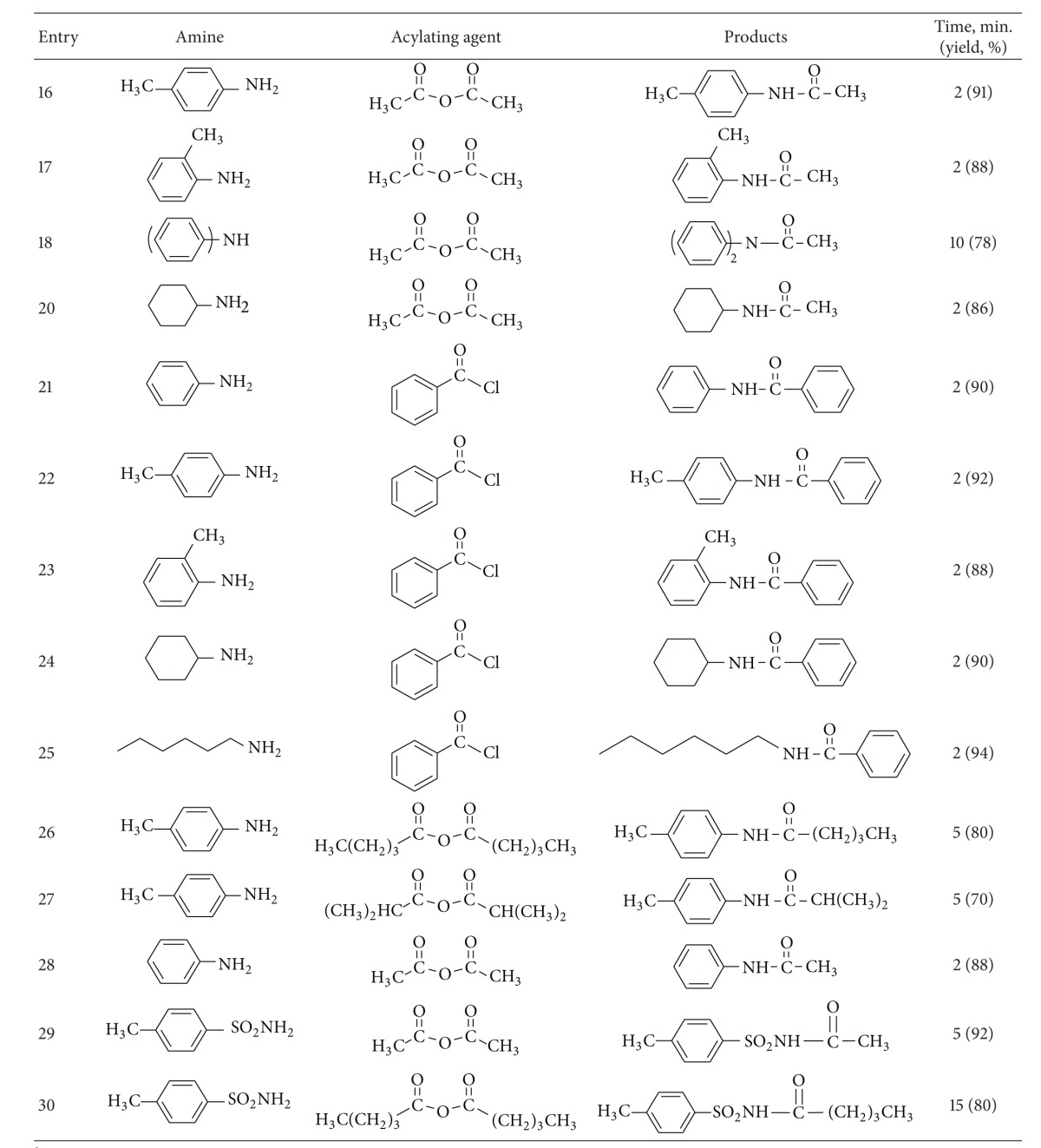

^a^20 mmol scale.

**Table 3:** ZSM-5-SO_3_H catalyzed acylation of alcohols and phenols under solvent-free conditions.

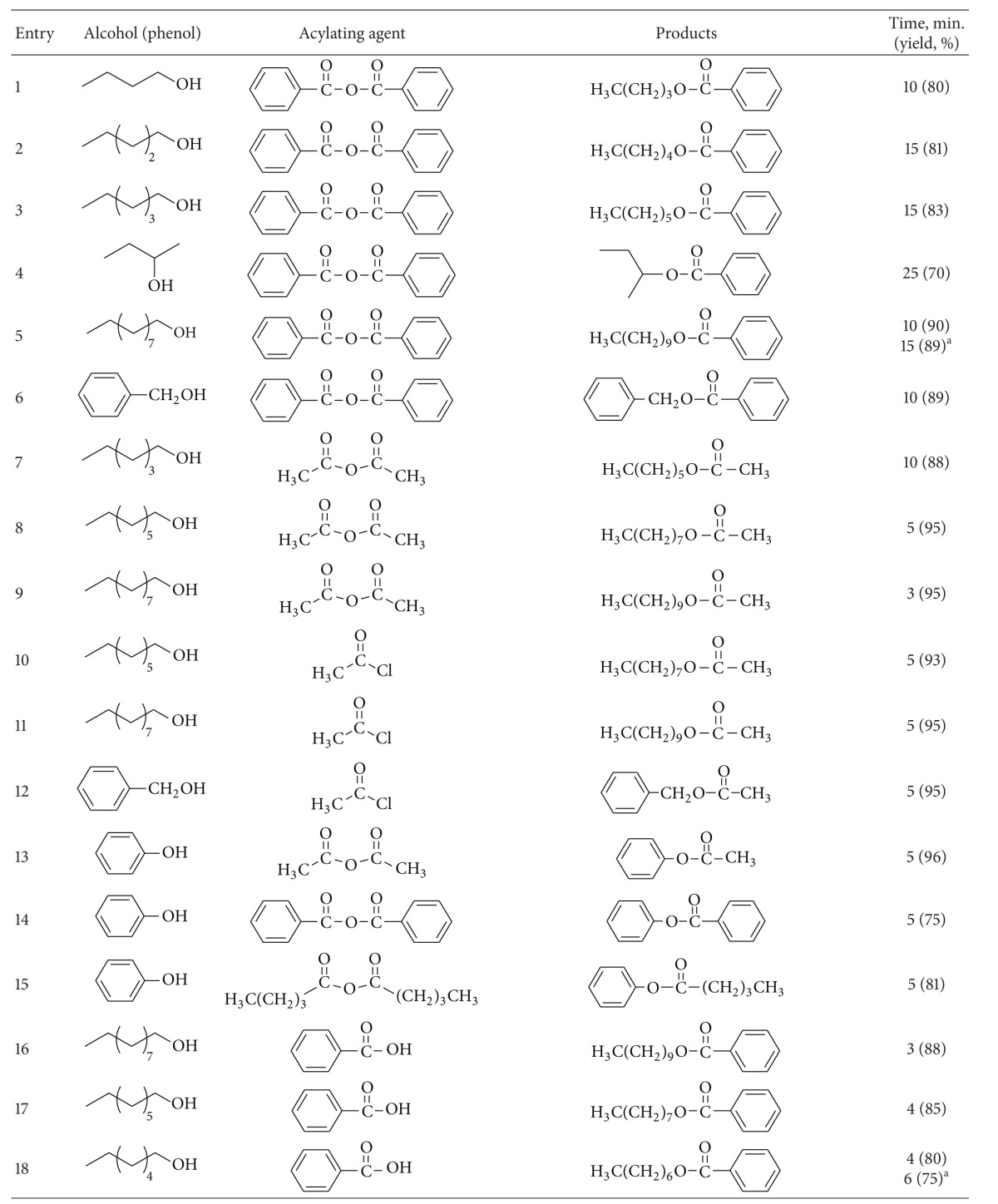 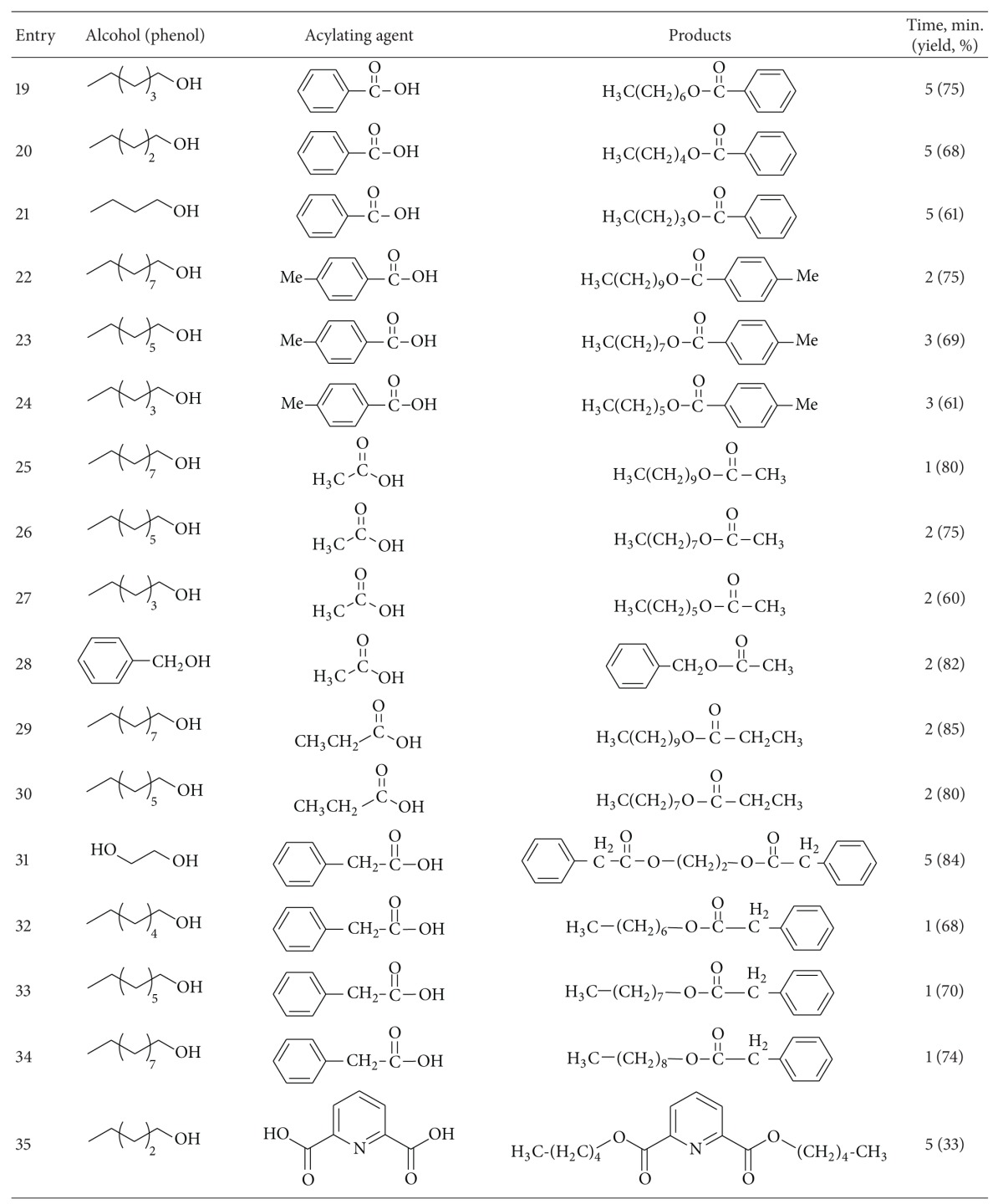 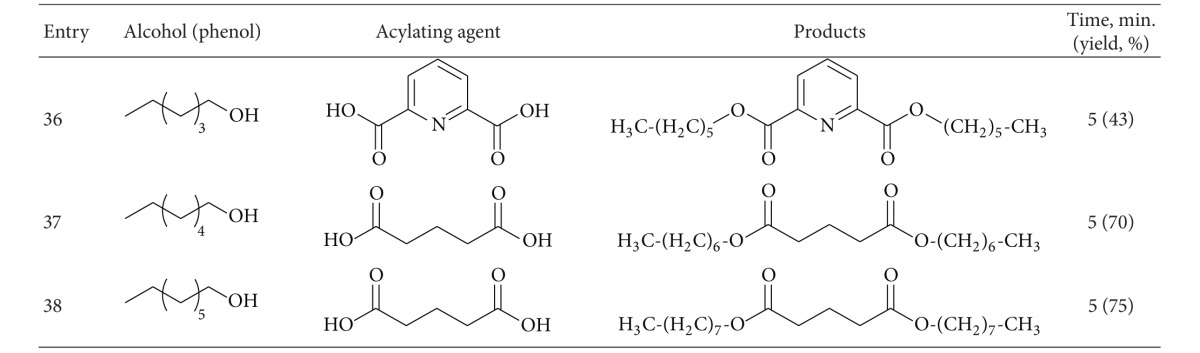

^a^20 mmol scale.
